# Ultrasound-guided bilateral erector spinae plane block in laparoscopic colon cancer surgery: A randomized controlled prospective trial

**DOI:** 10.1007/s00101-021-01076-6

**Published:** 2021-12-22

**Authors:** Qijin Li, Quanchu Li, Weiping Peng, Zhenzhen Liu, Yaohai Mai, Congying Shi, Ping Mo

**Affiliations:** 1 Department of Anesthesiology, Affiliated Nanhai Hospital of Southern Medical University, 40 Foping Road, 528200 Foshan, Guangdong China; 2 Department of Anesthesiology, The Sixth Affiliated Hospital, South China University of Technology, 528200 Foshan, Guangdong China; 3 Department of Experimental Center, Guangzhou Municipality Tianhe Nuoya Bio-engineering Co. Ltd, 510663 Guangzhou, Guangdong China

**Keywords:** ESPB, Oncology, ERAS, Rocuronium, Sensory extent, ESPB, Onkologie, ERAS, Rocuronium, Sensorisches Ausmaß

## Abstract

**Background:**

The efficacy of erector spinae plane block (ESPB) for pain control in other surgeries remains an interesting topic of discussion. This study aimed to evaluate the safety and efficacy and quality of recovery of ultrasound-guided bilateral ESPB in laparoscopic surgery for colon cancer.

**Material and methods:**

In this study 50 patients were included and randomly divided into the intervention group (E group, *n* = 25) and the control group (C group, *n* = 25). Patients in the E group received general anesthesia with preoperative bilateral ultrasound-guided ESPB, whereas patients in the C group received general anesthesia with saline injection in the erector spinae plane preoperatively. Data on intraoperative and postoperative anesthetic effects and the effect on enhanced recovery after surgery were recorded and analyzed.

**Results:**

Rocuronium consumption in the intervention group was 82.80 ± 21.70 mg, which was lower than that in the control group (*P* < 0.05). Visual analog scale scores at 2, 6, and 24 h after surgery in the intervention group were lower than those in the control group (*F*_*between*_ = 34.034, *P* *=* 0.000). The time to ambulation, consumption of ketorolac tromethamine, time to oral intake and hospital stay after operation in the intervention group were significantly lower than those in the control group* (P* *<* 0.05). The block area at the different baselines was significant (*F*_*between*_ *=* 3.211, *P* = 0.009). The association between baseline and time was significant (*F*_*baseline*_ _* time_ = 3.268, *P* = 0.001).

**Conclusion:**

This study confirmed that ultrasound-guided ESPB technology is safe and beneficial for patients with colon cancer undergoing laparoscopic colon surgery.

## Abstract (de)

**Hintergrund:**

Die Wirksamkeit des Erector-Spinae-Blocks (ESPB) zur Schmerzbekämpfung bei anderen Operationen bleibt ein interessantes Diskussionsthema. Diese Studie zielte darauf ab, die Sicherheit und Wirksamkeit sowie die Qualität der Genesung beim ultraschallgesteuerten bilateralen ESPB in der laparoskopischen Chirurgie bei Darmkrebs zu bewerten.

**Materialien und Methoden:**

In diese Studie wurden 50 Patienten eingeschlossen und nach dem Zufallsprinzip in die Interventionsgruppe (E-Gruppe, *n* = 25) und die Kontrollgruppe (C-Gruppe, *n* = 25) eingeteilt. Patienten in der E‑Gruppe erhielten eine Vollnarkose mit präoperativem bilateralem ultraschallgesteuertem ESPB, während Patienten in der C‑Gruppe präoperativ eine Vollnarkose mit Kochsalzinjektion in die Erector-Spinae-Ebene erhielten. Daten zu intraoperativen und postoperativen anästhetischen Effekten und der Wirkung auf eine verbesserte Genesung nach der Operation wurden aufgezeichnet und analysiert.

**Ergebnisse:**

Der Rocuroniumverbrauch in der Interventionsgruppe betrug 82,80 ± 21,70 mg, was niedriger war als in der Kontrollgruppe (*p* < 0,05). Visuelle Analogskalenwerte 2, 6 und 24 h nach der Operation in der Interventionsgruppe waren niedriger als in der Kontrollgruppe (*F*_*between*_ = 34,034, *P* = 0,000). Die Zeit bis zur Ambration, der Konsum von Ketorolac-Tromethamin, die Zeit bis zur oralen Einnahme und der Krankenhausaufenthalt nach der Operation in der Interventionsgruppe waren signifikant niedriger als in der Kontrollgruppe (*p* < 0,05). Die Blockfläche an den verschiedenen Basislinien war signifikant (*F*_*between*_ = 3,211, *p* = 0,009). Die Assoziation zwischen Baseline und Zeit war signifikant (*F*_*baseline*_ _* time_ = 3,268, *p* = 0,001).

**Schlussfolgerung:**

Diese Studie bestätigte, dass die ultraschallgesteuerte ESPB Technologie für Patienten mit Darmkrebs, die sich einer laparoskopischen Dickdarmoperation unterziehen, sicher und vorteilhaft ist.

## Introduction

Colorectal cancer is one of the most common tumors worldwide [1, 2]. The American Cancer Society estimated that 95,520 new cases of colon cancer were diagnosed in the United States in 2017 [3]. Both total mesorectal excision and complete mesocolic excision have become the standard techniques in colon cancer surgery to reduce local recurrence and mortality [4].

Improved standards in perioperative care can be attributed to a wide range of changes in clinical interventions. Enhanced recovery after surgery (ERAS) has been one of the most significant recent breakthroughs [5], particularly in patients undergoing abdominal surgery. The ERAS protocol is a multimodal, multidisciplinary, and evidence-based approach that reduces surgical stress, enhances early recovery after surgery, and improves patient outcomes [6]. Most ERAS protocols consist of perioperative, intraoperative, and postoperative care. Thus, the responsibility of the anesthetist is to modify the management to increase both the quality and outcome of perioperative care [7]. Regional anesthesia complements and enhances multimodal analgesia for abdominal surgery and improves surgical outcomes [8–11].

Thoracic epidural analgesia (TEA) and transversus abdominis plane (TAP) blocks are common interventions used in major open abdominal surgery but have certain disadvantages [12–15]. The ultrasound-guided erector spinae plane block (ESPB) was first described by Forero et al. [16] in 2016 for chronic and postoperative thoracic pain. Compared to TAP block, ESPB has the advantages of TEA as it has been reported to provide a wide range of sensory blockade in the abdomen [17–20] and thorax [21–25]. The ESPB also has a lower risk of complications than TEA or paravertebral blocks [16, 26, 27].

To evaluate the perioperative analgesic efficacy and the quality of recovery of ESPB in patients undergoing laparoscopic surgery for colon cancer, we conducted a randomized double-blind trial for laparoscopic colon cancer surgery to verify the safety and efficacy of ESPB. Its ERAS efficacy was also evaluated. This study quantified and assessed the block area in vivo using the temperature and prick test, after setting baselines for testing by assigning bones from T1 to L5.

## Methods

### Aim, design and participants

All procedures followed were in accordance with the ethical standards of the responsible committee on human experimentation and with the Declaration of Helsinki of 2000. Informed consent was obtained from all patients included in the study. The study was approved by the ethics committee of the Nanhai Hospital Affiliated to Southern Medical University (NO.: (2019) 531). This prospective randomized controlled double-blind study was registered in the Chinese Clinical Trial Registry (trial ID: ChiCTR2000031255, 26.03.2020). The study adhered to the Consolidated Standards of Reporting Trials (CONSORT) guidelines.

The inclusion criteria were as follows: (1) patients aged 18–75 years, (2) American Society of Anesthesiologists (ASA) physical status I–II, (3) New York Heart Association class I–II, (4) all patients scheduled for laparoscopic surgery for colon cancer, and (5) patient or family member who provided written informed consent. The exclusion criteria were as follows: (1) patients with abnormal coagulation function; (2) patients with severe heart and lung disease and liver and kidney dysfunction, (3) patients allergic to local anesthetic drugs or opioids, (4) patients receiving preoperative chemoradiotherapy, (5) patients unable to cooperate or communicate, (6) patients with chronic pain or a long history of mental illness, (7) patients with serious complications that were not caused by anesthesia that resulted in a prolonged hospital stay, (8) patients with any contraindications to regional anesthesia, and (9) patients who proactively withdrew from the study.

To evaluate the safety and efficacy and quality of recovery of ultrasound-guided bilateral ESPB in laparoscopic surgery for colon cancer, patients were assigned into two groups: the ESPB (E group) and control groups (C group). Patients in the E group received general anesthesia with preoperative bilateral ultrasound-guided ESPB. Patients in the C group received general anesthesia with preoperative bilateral ultrasound-guided erector spinae plane (ESP) saline injection.

### Interventions

Ultrasound-guided ESPB was performed under strict aseptic precautions. Each patient was placed in the right lateral decubitus position and underwent routine monitoring including electrocardiography; blood pressure, and pulse oximetry; and peripheral venous access. Invasive arterial blood pressure monitoring, central venous catheter placement right jugular internal vein, and catheterization in the right neck were performed. All the anesthesia operations were performed by the same experienced, skilled and senior attending physician. The T7 spinous process was located by direct palpation of the spinous processes starting from the C7 downward. The tip of the T7 transverse process was then identified using a linear array high-frequency ultrasound probe placed in a transverse orientation. By rotating the ultrasound probe to a portrait orientation, a parasagittal view of the skin, subcutaneous tissue, trapezius, and erector spinae muscle was displayed. The T7 transverse process could be confirmed when the rhomboid muscle disappeared because the rhomboid muscle was located at the T5–T6 vertebral level. To confirm correct needle insertion, the needle was inserted in-plane craniocaudally until it reached the T7 transverse process and 0.5–1.0 mL saline was injected. After the injection the distribution of the injected saline was assessed in order to verify the correct needle position and injection plane. No distension observed in the erector spinae muscle meant that the tip of the needle was in the correct plane. Then, patients in the E group were injected with 20 mL of ropivacaine (0.25%) into the ESP bilaterally. Patients in the C group were injected with 20 mL of saline bilaterally. All the injections were performed in the correct plane. Both groups received general intravenous anesthesia by subsequently injecting 0.4 μg/kg sufentanil, 2 mg/kg propofol, and 0.6 mg/kg rocuronium. Tracheal intubation was performed after muscle relaxation. Mechanical ventilation was then performed and the tidal volume was set to 6–8 mL/kg and respiratory rate to 12–16 times/min. Moreover, end-tidal carbon dioxide was maintained at 35–45 mm Hg. A bispectral index (BIS) monitor was used to monitor anesthesia depth, BIS values were controlled to 46–60 and mean arterial blood pressure was maintained at 70–100 mm Hg. A dual channel target-controlled infusion pump (WeiLiFangZhou TCI-III‑B [Nan Ning, Guang Xi, China]) was used to infuse propofol, remifentanil and rocuronium for maintaining anesthesia during surgery. The propofol was infused 1–4 μg/mL in the Marsh mode and remifentanil was infused 2–4 ng/mL in target-controlled infusion with effect-site concentration. The dosage of rocuronium can be adjusted according to the surgeon’s need for muscle relaxation during surgery. An electronic intravenous analgesia pump was used in both groups postoperatively. Sufentanil (2 μg/kg) and tropisetron (4 mg) were diluted in 100 mL of saline for the analgesia pump. The pump infusion rate was 2 mL/h, with a single patient-controlled intravenous analgesia dose of 0.5 mL and duration of 15 min. Ketorolac tromethamine (30 mg per dose) was administered as an analgesic remedy when pain became unbearable (visual analog scale [VAS] > 5).

**Fig. 1:**
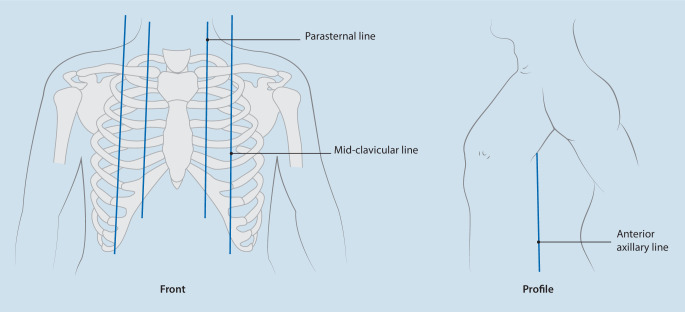
Baselines of the temperature and prick test

The temperature and prick test were used separately to detect the temperature by alcohol evaporation and pain sensory extent of the block after surgery. The left parasternal line, left mid-clavicular line, left anterior axillary line, right parasternal line, right mid-clavicular line, and right anterior axillary line were used as baselines (Fig. 1).

### Outcome measures

Postoperative pain was assessed using the VAS at 2, 6, 24, and 48 h postoperatively. A rescue analgesic was infused when the patient had a VAS score > 5, and the total analgesic consumption of ketorolac tromethamine was recorded within 48 h. Arterial blood was collected upon admission to the operating room (Time 1), 10 min after extubation (Time 2), and 24 h after operation (Time 3), and stress response indices such as blood glucose and lactate were then detected. Components of ERAS, such as the first time of anal exhaust, the time of leaving the bed, eating, bladder catheter removal, and hospital stay, were recorded. Data regarding the infusion of sufentanil and remifentanil during surgery were recorded. The numbers of pressing in the analgesia pump within 48 h postoperatively were recorded, as were the postoperative complications such as bradycardia, hypotension, nausea, vomiting, pruritus, respiratory depression or vertigo. The sensory extent of the block was noted at 6, 12, and 24 h postoperatively. The L5-T1 bones were assigned as variables 1–17 according to the order of the lumbar vertebrae to the thoracic vertebrae from bottom to top.

### Statistical analyses

All surgeons, nurses, follow-up data collection doctor, statisticians, and patients were blinded to group allocation. Only the anesthesiologists performing the blocks and operating room nurses were not blinded. The surgeons who performed the colon cancer surgery were blinded to the group allocation. None of the anesthesiologists involved in the study followed up the patient in the postoperative period and collected data for the study. The follow-up data were collected when patients were hospitalized by a designated doctor. Ward nurses in the postoperative period were blinded to the group allocation. Qijin Li, Quanchu Li, Weiping Peng, Zhenzhen Liu, and Yaohai Mai enrolled participants. Participants were assigned to groups E and C through random sampling method by Doctor Ping Mo. The E and C groups were assigned as “A” and “B” by Doctor Ping Mo. The statistician was blinded when analyzing. The groups were blinded until all data collection was complete.

A previous trial found that the mean ± standard deviation of VAS pain score at 24 h postoperatively in patients with ESPB was 2.76 ± 0.68. We hypothesized a common within-group standard deviation of 0.7 and calculated that a sample size of 25 patients per group (total 50) would provide 80% power at a two-sided α value of 0.05 to detect the difference in VAS pain score at 24 h postoperatively between patients in the E and C groups. To account for possible protocol violations, we enrolled a total of 80 participants.

Descriptive analysis was used to analyze the characteristics of the selected patients. Baseline clinical characteristics of the two groups were compared using the χ^2^-test. Mean values and standard deviation were calculated and determined using a t-test for each quantitative variable. Repeated measures analysis of variance (ANOVA) was performed to compare repeated measures variables. The variable model area was calculated, and repeated measures ANOVA was used to evaluate anesthesia efficacy. Statistical significance was set at *P* < 0.05. All data were analyzed using the PASW Statistics for Windows version 18.0 statistical software (Chicago: SPSS Inc., 2009).

## Results

### Patient characteristics

**Fig. 2:**
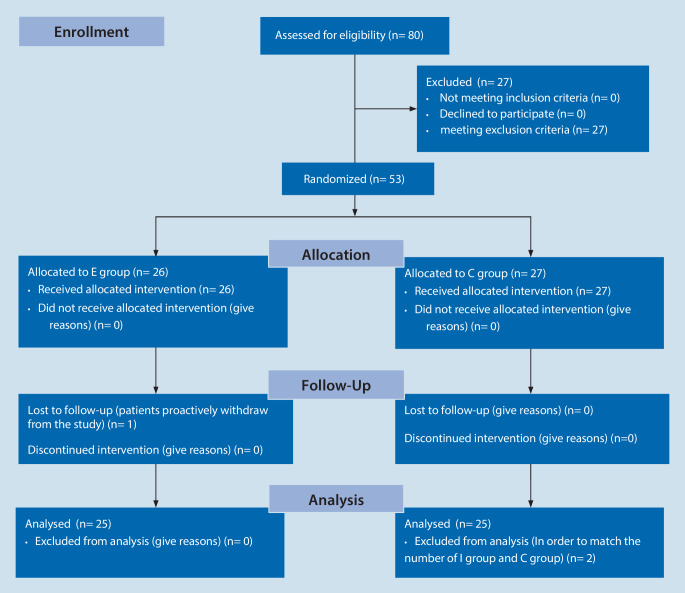
CONSORT flow diagram

The study recruited and followed up participants from 1 July 2019, to 31 August 2020. During the study period, 80 patients were considered eligible; however, 27 patients were excluded for meeting the exclusion criteria. A total of 53 patients were finally included and were randomized and 27 patients received general anesthesia with preoperative saline injection (C group), whereas the other 26 patients received ESPB in addition to general anesthesia (E group). One patient from the E group withdrew from the study during the follow-up period. To match the number of the E group and the C group, two patients in the C group were randomly excluded. The CONSORT flow diagram is shown in Fig. 2. There was no significant difference between the demographics of patients in the control group and the intervention group (Table 1).

**Table 1:** Comparison of patient demographic data

	E group (*n* = 25)	C group (*n* = 25)	*P*
Sex (male:female)	15:10	15:10	1.000
Age (years)	62.48 ± 10.96	67.89 ± 10.89	0.087
Body mass index (kg/m^2^)	21.28 ± 2.96	20.35 ± 3.51	0.333

### Outcomes

The consumption of rocuronium in the E group was 82.80 ± 21.70 mg, which was significantly lower than that in the C group (*P* *<* 0.05); however, there was neither a statistically significant difference between the dosages of remifentanil and propofol (Table 2) nor in the amount of lactic acid in the blood between the two groups (*F*_*between*_, *P* *>* 0.05). The associations between lactate and glucose in the blood over time were not significant in the two groups (*F*_*group*_ _* time_*, P* *>* 0.05) (Table 3).

**Table 2:** Comparison of anesthetic dosage during surgery

	E group (*n* = 25)	C group (*n* = 25)	*P*
Propofol (mg)	1056.00 ± 445.35	1028.80 ± 473.68	0.835
Remifentanil (μg)	1209.20 ± 364.36	1082.76 ± 420.70	0.262
Rocuronium (mg)	82.80 ± 21.70	116.60 ± 48.38	0.003

**Table 3:** Comparison of blood glucose and lactate at different detection times, postoperative VAS score, and ERAS efficacy

	E group (*n* = 25)	C group (*n* = 25)	*F*_*within*_	*F*_*between*_	*F*_*group*_ _* time_	*P*
*Blood glucose (mmol/L)*						
T1	6.53 ± 2.53	6.60 ± 1.54	6.953^**^	0.047	0.151	–
T2	7.33 ± 1.96	7.59 ± 1.74				–
T3	7.19 ± 2.82	7.20 ± 1.48				–
*Blood lactate (mmol/L)*						
T1	1.00 ± 0.59	0.65 ± 0.40	29.770^**^	3.175	0.362	–
T2	1.41 ± 0.71	1.14 ± 0.63				–
T3	1.36 ± 0.84	1.12 ± 0.45				–
*VAS score*						
2 h after surgery	2.12 ± 0.53	2.32 ± 0.75	32.801^**^	10.634^**^	7.021^**^	–
6 h after surgery	2.72 ± 0.61	3.68 ± 0.63				–
24 h after surgery	2.76 ± 0.83	3.32 ± 0.75				–
Time to ambulation (h)	30.40 ± 10.20	51.04 ± 14.39	–	–	–	0.000
Analgesia remediation consumption of ketorolac tromethamine (mg)	24.00 ± 35.71	51.60 ± 43.75	–	–	–	0.018
Anal exhaust time (h)	53.44 ± 18.29	60.64 ± 16.19	–	–	–	0.147
Time of bladder catheter removal (h)	72.00 ± 142.32	54.08 ± 33.94	–	–	–	0.543
Time to oral intake (h)	36.96 ± 17.97	52.00 ± 16.45	–	–	–	0.003
Hospital stay after operation (days)	10.08 ± 5.05	12.52 ± 2.60	–	–	–	0.037

*P < 0.05, **P < 0.01

The VAS scores at 2, 6, and 24 h after surgery in the E group were lower than those in group C (*F*_*between*_ = 34.034, *P* *=* 0.000). Furthermore, the association between anesthesia methods and VAS time was statistically significant between the two groups (*F*_*group*_ _* time_ = 9.759, *P* = 0.000) (Table 3), indicating that ESPB anesthesia method had a longer analgesic effect. For ERAS, the time of leaving the bed, analgesia remediation consumption of ketorolac tromethamine, meal time after surgery and hospital stay in the E group were significantly lower than those in the C group (*P* *<* 0.05). Differences in parameters such as anal exhaust time and time of bladder catheter removal between groups were not statistically significant (*P* *>* 0.05) (Table 3).

The incidences of postoperative complications are shown in Table 4. The incidences of postoperative complications in E group were lower than that in C group, but not significant different (*P* *>* 0.05).

**Table 4:** Comparison of postoperative complications

	Postoperative complications *n* (%)						
	Bradycardia	Hypotension	Nausea and vomiting	Vertigo	Pruritus	Respiratory depression	Total
E group (*n* = 25)	0 (0)	0 (0)	1 (4)	0 (0)	0 (0)	0 (0)	1 (4)
C group (*n* = 25)	0 (0)	0 (0)	2 (8)	1 (4)	0 (0)	0 (0)	3 (12)
*χ*^*2*^	–	–	–	–	–	–	1.087
*P*	–	–	–	–	–	–	0.297

### Sensory extent of the block

**Fig. 3:**
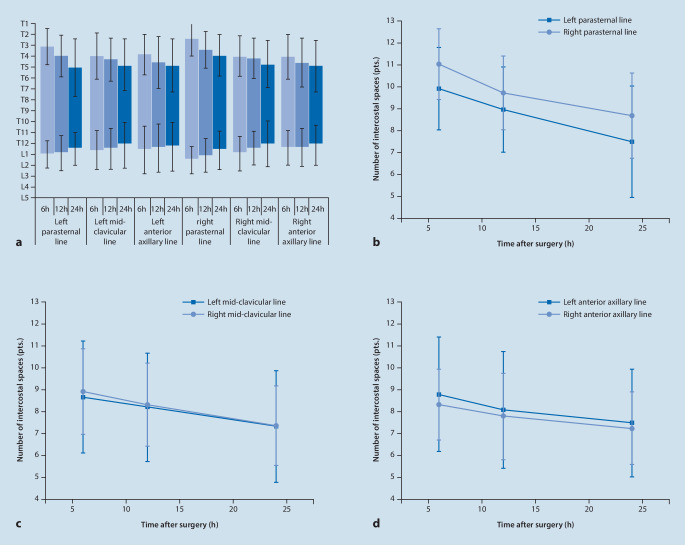
Sensory extent of the erector spine muscle block after surgery. **a** The extent of the sensory block shown by the vertebral column; **b** The block extent of parasternal line is shown in number of intercostal spaces; **c** The block extent of mid-clavicular line is shown in number of intercostal spaces; **d** The block extent of anterior axillary line is shown in number of intercostal spaces

The extent of the sensory block was determined by the temperature and prick test for the E group (Fig. 3a). Over time, the extent of the block gradually decreased (*F*_*within*_ *=* 154.128,* P* *=* 0.000). The difference in the blocked areas using the different baselines was significant (*F*_*between*_ *=* 3.211,* P* *=* 0.009). The association between baseline and time was significant (*F*_*baseline*_ _* time_ = 3.268, *P* = 0.001), indicating that the extent of the sensory block diminished over time (Table 5; Fig. 3b–d).

**Table 5:** Repeated measures ANOVA of block area

	6 h after surgery	12 h after surgery	24 h after surgery
Left parasternal line	9.92 ± 1.89	8.96 ± 1.95	7.52 ± 2.57
Left mid-clavicular line	8.68 ± 2.56	8.20 ± 2.48	7.36 ± 2.60
Left anterior axillary line	8.80 ± 2.61	8.08 ± 2.68	7.48 ± 2.47
Right parasternal line	11.00 ± 1.63	9.72 ± 1.67	8.68 ± 1.95
Right mid-clavicular line	8.92 ± 1.96	8.32 ± 1.91	7.36 ± 1.82
Right anterior axillary line	8.32 ± 1.63	7.80 ± 1.98	7.28 ± 1.70
*F*_*within*_	154.128^**^		
*F*_*between*_	3.211^**^		
*F*_*baseline*_ _* time_	3.268^**^		

*P < 0.05, **P < 0.01

## Discussion

As a new blocking technique, the use of erector spinal plane block has been reported in mammary glands, thoracoscopic and spinal surgery [28]. ESPB is a promising regional anesthesia technique. Physicians can reach proficiency in performing these blocks in a shorter time than other more invasive techniques such as paravertebral blocks; however, there are only a few randomized controlled double-blind studies on abdominal surgery.

Our results showed that the consumption of rocuronium in E group was significantly lower than that of C group. We reviewed all the surgical records in this study and found that 5 patients in the control group received an additional dosage of 0.3 mg/kg rocuronium due to intraoperative body movements or dissatisfaction with the effect of muscle relaxation but no such situation occurred in the experimental group. We speculate that is the main reason why the rocuronium consumption between two group is significant different, and ESPB could provide a better muscle relaxation. The consumption of propofol and remifentanil during operation between two groups were not significantly different. This could be attributed to TCI target-controlled remifentanil under the guidance of ERAS, which lead to a relatively stable circulation and no drastic hemodynamic fluctuation. Glucose and lactate levels in the blood were not significantly different between the two groups at different times, indicating that ESPB has no evident inhibitory effect on the stress injury induced by surgery. The same remifentanil consumption in both groups indicated that the ESP block did not produce visceral analgesia but both groups were given analgesic pump after operation, which had a certain effect on visceral pain. The bilateral ultrasound-guided ESPB resulted in a significant postoperative reduction of pain through the VAS scores at 2, 6, and 24 h. The difference in VAS may be due to the pain of the wound, and ESPB can provide better analgesia. The numbers of nausea, vomiting and vertigo in the C group were higher than in E group. It was possible that the number of postoperative salvage analgesia and analgesia pump compressions in the C group were more than that in the E group, resulting in increased adverse reactions. This may also indicate that the analgesic effect of ESP in E group was better than C group. We had also assessed the VAS scores of patients in two groups at 48 h. The VAS score of the E group at 48 h was 2.40 ± 0.76 and that of the C group was 2.32 ± 0.56. The VAS score data of 48 h were both low without significant difference; thus, we did not analyze it in the results. The time to ambulation, analgesia remediation consumption of ketorolac tromethamine, time to oral intake and hospital stay after operation in the E group were significantly reduced, indicating that the effects of ESPB on ERAS were better than the C group. The concept of ERAS is popular in gastrointestinal surgery. The ESPB anesthesia can effectively reduce postoperative pain and postoperative complications in patients, meeting the technical requirements of ERAS. The results of our investigations in the extent of sensory block showed that the ranges of anesthesia within 24 h in different baselines were both between T12 and T5. The left and right parasternal anesthesia lines were more extensive than others, but the effect of anesthesia declined more rapidly. The anesthetic effect in the right side of the body was stronger than the left side, which may be related to the lateral position during performance of the ESPB; however, the range of anesthesia for 24 h was between T12 and T5. The declines in the sensory extent of the block at 12 h and 24 h were 8.67% and 18.2%, respectively. In addition, No ESPB-related complications occurred (hematoma etc.), no ESPB failed due to technical difficulties, proving that ESPB under ultrasound guidance is safe and effective. Although the history of ESPB is short, and few controlled clinical trials have been published, there is an abundance of case reports. Some dealt with analgesia for abdominal surgery [19, 29, 30], but not for laparoscopic surgery for colon cancer and quality of recovery. Our prospective randomized controlled trial has confirmed that ultrasound-guided ESPB technology is safe and beneficial for laparoscopic colon surgery for colorectal cancer.

In this study, we found that the standard deviation of the block area was large, suggesting that the block area of ESPB may be significantly different among individuals. ESPB was placed at T7 in this study and the block spread to T1, in contrast to another study [31]. Cadaver studies examining local anesthetic spread post-ESPB have shown inconsistent dye spread [32], whereas our data showed that the block area at different baselines did not significantly differ.

Our study has some limitations. First, this was a single-center study. The number of participants enrolled was small, so the applicability of results may be limited. Second, there was no effective evaluation index used in this study to distinguish visceral pain from incisional pain, which needs to be confirmed by further studies. Third, the mechanism of ESPB should be further studied by exploring the diffusion pathway after drug injection by adding a contrast agent to local anesthetics. Fourth, our study found that some patients still have pain after surgery > 24 h. Dexamethasone or dexmedetomidine can be added to the anesthetics to prolong block time in further studies. Finally, the recovery quality scale, 15-item quality of recovery and other relevant evaluation indicators can be used to evaluate the quality of the patient recovery and postoperative status.

In conclusion, our prospective randomized controlled trial has confirmed that ultrasound-guided ESPB technology is safe and beneficial and improves recovery rate and ERAS in patients with colon cancer undergoing laparoscopic colon surgery.

## Abbreviations

BIS: Bispectral index
CME: Complete mesocolic excision
ERAS: Enhanced recovery after surgery
ESP: Erector spinae plane
ESPB: Erector spine plane block
TAP: Transversus abdominis plane
TEA: Thoracic epidural analgesia
TME: Total mesorectal excision
VAS: Visual analog scale
